# Evaluating discrimination of prediction models for recurrent clinical events

**DOI:** 10.1186/s41512-026-00238-7

**Published:** 2026-09-07

**Authors:** Thomas J. Spain, Alexandra Hunt, Maria Sudell, Jane L. Hutton, Hein Putter, Victoria Watson, John Blakey, Anthony Marson, Laura Jayne Bonnett

**Affiliations:** 1 Department of Health Data Science, University of Liverpool, Waterhouse Building, Block F, 1-5 Brownlow Street, Liverpool, L69 3GL UK; 2 Department of Statistics, Zeeman Building, University of Warwick, Coventry, CV4 7AL UK; 3 Leiden University Medical Center, University of Leiden, Albinusdreef 2, Leiden, 2333 ZG Netherlands; 4 Phastar, 2D Bollo Ln, Chiswick, London, W4 5LE UK; 5 Respiratory Medicine, Sir Charles Gairdner Hospital, Perth, WA 6009 Australia; 6 Medical School, Curtin University, Perth, WA 6102 Australia; 7 Department of Pharmacology & Therapeutics, University of Liverpool, Liverpool, L69 7BE UK

**Keywords:** Prediction, Recurrent events, Discrimination

## Abstract

**Background:**

Prediction models for clinical conditions involving recurrent events require performance metrics tailored to repeated outcomes. Although we and others have developed methods to evaluate calibration and related aspects of performance, approaches for assessing discrimination remain limited. Recent contributions, including concordance-based methods and Brier-type accuracy measures, address parts of this gap, but no widely adopted, flexible framework for discrimination exists. As discrimination is a core TRIPOD+AI–recommended metric, accessible methodology and software for evaluating how well recurrent event models separate higher- and lower-risk individuals are still needed.

**Methods:**

We propose adapted discrimination statistics based on comparing predicted and observed cumulative event counts, addressing limitations of conventional concordance approaches. Statistical uncertainty is evaluated using several resampling strategies, including delete-d jackknifing, with user-friendly R code provided. We illustrate the approach using four tie-handling methods (c statistic, Kendall’s τₐ, Somers’ D, Goodman–Kruskal’s γ), four recurrent event models (negative binomial, zero-inflated negative binomial, Andersen–Gill, and Prentice–Williams–Peterson Total Time), and two clinical datasets: the SANAD epilepsy trial and an OPCRD asthma cohort.

**Results:**

Discrimination varied substantially across models. In the asthma data, the Prentice–Williams–Peterson model showed the strongest discrimination (C = 0.939, 95% CI 0.935–0.944), with rank-based metrics (τₐ = 0.603; Somers’ D and γ = 0.879) also indicating very strong discrimination. The Andersen–Gill model performed moderately well (C = 0.823, 95% CI 0.815–0.830), while the negative binomial (C = 0.573) and zero-inflated negative binomial models (C = 0.586) showed weak discrimination. In the epilepsy data, the Prentice–Williams–Peterson model again performed best (C = 0.943, 95% CI 0.912–0.974), with all rank-based metrics above 0.85. The negative binomial, zero-inflated negative binomial and Andersen–Gill models showed moderate discrimination.

**Conclusion:**

This study provides practical methods and open-source code for evaluating the discrimination of prediction models for recurrent event data. Used alongside existing calibration tools, these methods support comprehensive performance evaluation and help standardise validation and reporting for prediction models developed for recurrent clinical events as recommended by the TRIPOD + AI guidelines.

**Supplementary Information:**

The online version contains supplementary material available at https://doi.org/10.1186/s41512-026-00238-7.

## Background

Clinical prediction models are essential tools in healthcare, informing diagnosis, prognosis, and therapeutic decision-making. They combine multiple pieces of patient information to predict a clinical outcome for people with an underlying health condition [1]. When developing a clinical prediction model, it is important to evaluate the model’s calibration and discrimination [2]. Calibration evaluates whether the probability of the outcome predicted by the model agrees with the observed probability of the outcome. Discrimination considers how well the model is able to differentiate between those individuals who have the outcome, and those that do not [1]. 

Many prediction models are developed and published each year [3]. They are frequently developed using logistic regression or time to event approaches and increasingly via machine learning methods [4]. Methodology and computer code exist to evaluate discrimination and calibration of models developed using these approaches [5]. However, many medical conditions are typified by repeated episodes of the same type; repeated seizures for people with epilepsy or repeated exacerbations for people with asthma for example. Such events can be included within prediction models via modifications to the Cox model such as Andersen-Gill [6] and Prentice, Williams and Peterson models [7]. Shared Gamma Frailty models have also been identified as useful approaches for this type of data [8]. In addition to recurrent event survival models, count-based approaches such as Negative Binomial (NB) and Zero-Inflated Negative Binomial (ZINB) models have also been proposed for analysing recurrent event outcomes [9]. These approaches typically focus on modelling the overall event rate or cumulative event burden during follow-up and may account for differing observation times through offset terms. In contrast, recurrent event survival models explicitly incorporate the timing and ordering of recurrent events within a hazard-based framework, naturally accommodating censoring and variable follow-up durations. Some approaches additionally allow event-specific baseline hazards or time-varying covariate effects [8]. For example, Andersen–Gill (AG) models extend the Cox proportional hazards framework using a counting-process approach with a common baseline hazard across recurrent events, whereas Prentice, Williams and Peterson (PWP) models stratify according to event order and allow the baseline hazard to vary between successive events [10]. However, PWP models may become unstable for higher-order events when relatively few individuals remain under observation in later event strata [8]. 

Whilst methodology is available to evaluate calibration and model fit of recurrent event prediction models [11], limited approaches are available to evaluate discrimination. We therefore build on existing concordance and rank-based approaches by adapting multiple discrimination statistics to recurrent events and by providing a resampling framework, including delete-d jackknifing, to quantify statistical uncertainty.

This paper, therefore, addresses this gap in the literature by proposing methodology to easily assess discrimination for recurrent event data. The methodology is implemented in R code and applied to two clinical examples, epilepsy and asthma, and demonstrated across several commonly used recurrent event model types. The R code is made publicly available to ensure that all researchers can fully evaluate the performance of recurrent event prediction models in future.

## Methods

In this work, the observed outcome for each individual is defined as the cumulative number of events experienced during a specified follow-up period, for example the number of asthma exacerbations within one year of study entry. The prediction from a recurrent event model is the expected cumulative number of events over the same time horizon.

### Discrimination for recurrent event prediction models

To evaluate calibration for prediction models with recurrent events, predicted and observed probabilities of the outcome as used in traditional measures [1] can be replaced with predicted and observed cumulative event counts [11]. We apply a similar logic here to modify existing methodology to evaluate discrimination for prediction models with recurrent events.

The existing calculation of discrimination considers paired data values, $\:({x}_{i},{y}_{i})$, where $\:{y}_{i}$ is the observed data for individual * i*, and $\:{x}_{i}$ is the prediction for individual * i*. The prediction is the predicted probability of the outcome (e.g. death or remission from seizures), at a specified time point if the prediction model has been built using a time-to-event approach. A pair of observations * (a,b)* is considered concordant if the prediction and the observed value are in the same direction, i.e. $\:({x}_{a}\mathrm{>}{x}_{b},{y}_{a}\mathrm{>}{y}_{b})$ or $\:({x}_{a}\mathrm{<}{x}_{b},{y}_{a}\mathrm{<}{y}_{b})$. In real terms this means that the person with the higher predicted probability of the outcome in the pair experienced the event before the person with the lower predicted probability of the outcome. If the prediction and observed value are not in the same direction, the pair is classed as discordant. The concordance statistic, * c*, is the proportion of concordant pairs across all possible pairings. There are many variants on the concordance statistic currently used within the literature. These are defined below and vary mainly by how any tied events are handled.

Tied values can occur in either the observed values or the predicted values. A tie is not counted as concordant because it provides no evidence that the model correctly ranked the risk between two individuals. However, tied pairs can be considered as incomparable, as discordant, or given a score of 0.5. The most conservative approach is Kendall’s $\:{\tau}_{a}$, which is shrunk towards zero when ties are present by considering ties as discordant. The formula is:

$$\:{\tau\:}_{a}\mathrm{=}\frac{con\mathrm{-}dis}{con\mathrm{+}dis\mathrm{+}{t}_{x}\mathrm{+}{t}_{y}\mathrm{+}{t}_{xy}}$$

where * con* is the number of concordant pairs, * dis* is the number of discordant pairs, $\:{t}_{x}$ is the number of pairs tied on the prediction, $\:{t}_{y}$ is the number of pairs tied on the observation (but not the prediction), and $\:{t}_{xy}$ is the number of pairs tied on both the prediction and the observation. These definitions are used throughout this manuscript.

In the recurrent event setting we treat the total number of events observed within the chosen time horizon as the outcome used to rank individuals. Therefore, for any pair of individuals * i* and * j*:

- If the individual with the higher predicted event count also has the higher observed event count, the pair is concordant.
- If the ordering is reversed, the pair is discordant.
- If either the predicted or observed counts are equal, the pair is treated as a tie according to the chosen statistic.

Kendall’s $\:{\tau}_{a}$ represents the strength of association between the predicted probabilities and observed outcomes, but penalises the measure heavily for tied values. Kendall’s $\:{\tau}_{a}$, Goodman-Kruskal’s $\:\gamma\:$ and Somer’s D range from − 1 to 1, while *c* ranges from 0 to 1. A $\:{\tau}_{a}$, $\:\gamma\:$ and D value near 1 indicates strong agreement between predicted risks and observed outcomes, while a value near − 1 suggests strong disagreement. A value close to 0 indicates no association. As it accounts for all types of ties, $\:{\tau}_{a}$ tends to be more conservative than other measures.

The Goodman-Kruskal $\:\gamma\:$ statistic ignores ties in either the prediction or outcome:

$$\:\gamma\:\mathrm{=}\frac{con\mathrm{-}dis}{con\mathrm{+}dis}$$

Goodman-Kruskal’s $\:\gamma\:$ measures the association between the predictions and the outcomes without considering any ties. It captures the strength and direction of association based only on concordant and discordant pairs. As it disregards tied pairs, $\:\gamma\:$ can appear artificially high when ties are frequently occurring in the data [12]. 

Somers’ * D* is the most frequently reported version of discrimination in statistical software [12], and can be easily converted to the concordance statistic, * c*, which is easier to interpret [1]. It treats the observed ties as incomparable and pairs that are tied in the predictions (not the observations) are scored as 0.5:

$$\:c\mathrm{=}\frac{\left(D\mathrm{+}1\right)}{2}\mathrm{=}\frac{con\mathrm{+}\frac{{t}_{x}}{2}}{con\mathrm{+}dis\mathrm{+}{t}_{x}}$$

Somers’ * D* measures the difference between the probability of concordance and discordance, while partially accounting for ties in predictions. It reflects how well the model can separate individuals with different outcomes, discounting outcome ties. It strikes a balance between conservativeness (like $\:{\tau\:}_{a}$) and ignoring ties (like $\:\gamma\:$). For the * c* statistic, a value of 0.5 corresponds to flipping a coin to make a prediction for each individual.

For recurrent event prediction models, we again modify the observed and predicted event probabilities with cumulative observed and predicted event counts as follows.

1. Evaluate the predicted total event count from study entry/start time to a pre-specified prediction horizon (e.g. 1 year or 2 years) for every individual in the dataset.
2. Consider each possible pairing of individuals.
3. If the subject in the pair with the higher predicted total event count by the chosen time point was observed to have the higher total event count, then the pair is considered concordant. This is irrespective of the magnitude of the difference in predicted counts between the pairs as concordance is a ranking measure, not a measure of how far apart predictions are [12].
4. If the subject in the pair with the higher predicted total event count by the chosen time point was observed to have the lower total event count, then the pair is considered discordant irrespective of the magnitude of the difference in predicted counts between the pairs.
5. If there is tied data, then consider the nature of the tie, $\:{t}_{x}$, $\:{t}_{y}$, or $\:{t}_{xy}$.
6. Calculate the concordance according to the formula for either Kendall’s $\:{\tau}_{a}$, Goodman-Kruskal $\:\gamma\:$, Somers’ * D* or * c* according to researcher preference. It is typical to include a single metric in an analysis.

The proposed discrimination measures were evaluated within pre-specified prediction horizons, with both predicted and observed recurrent event quantities restricted to the same follow-up window for each individual. Consequently, the resulting concordance measures should be interpreted as fixed-horizon assessments of discrimination for cumulative recurrent event burden within the specified prediction period.

### Associated measures of spread

Confidence intervals for discrimination are frequently estimated via bootstrap resampling [2]. However, when discrimination statistics involve comparisons of event counts rather than probabilities, tied values occur substantially more often. These ties reduce the number of informative pairwise comparisons and consequently lead bootstrap procedures to underestimate the variability of the discrimination statistic [12]. 

Similar challenges arise for models such as the PWP-Gap Time (PWP-GT) model. The PWP-GT model resets the time scale following each recurrent event and therefore models the elapsed time between successive events, whereas the PWP-TT model uses total follow-up time from study entry as the underlying time scale. Within the PWP-GT model, if two gap times within a patient are identical by chance, the model is unable to treat them as distinct events, resulting in loss of information and further reducing the number of meaningful comparisons available for variance estimation [13]. In light of these limitations, and motivated by previous methodological work recommending jackknife procedures for pairwise agreement measures [14], we recommend using jackknife resampling rather than bootstrapping to quantify uncertainty in discrimination estimates for recurrent event prediction models.

Jackknife resampling is similar to bootstrapping. However, rather than taking a user-defined number of samples of the same size as the original dataset $\:\left(n\right)$, with replacement, jackknifing recreates * n* new samples, each of size $\:n\mathrm{-}1$ by omitting one observation from the original sample [15]. The approach respects the correlation structure of the recurrent events by treating the patient, not the event, as the unit of deletion.

Estimation of confidence intervals for discrimination statistics depends not only on the total sample size but also on the number of informative, non-tied comparisons between individuals. To estimate the confidence intervals, for each jackknife sample the discrimination is calculated using the researcher’s preferred method from those described in Section  "Discrimination for recurrent event prediction models". These are called jackknife replicates, written as $\:{jk}_{i}$ for $\:i\text{}\in\:\text{}1:n$.

In general, discrimination measures behave as U-statistics, for which the sampling distribution becomes approximately normal with sufficiently large samples [16]. However, in the presence of a high proportion of tied predictions or outcomes, common in recurrent event data, the effective sample size is reduced, impairing the validity of normal approximations for confidence intervals. Empirical studies and simulation work for traditional (non recurrent event prediction models) suggest that at least 200–300 individuals and 100–150 events are required for stable estimation of discrimination confidence intervals under ideal conditions, with substantially larger samples (e.g., > 500) necessary when event rates are low, censoring is heavy, or ties are frequent [16–18]. 

If the sample size is large enough, it might be appropriate to use a normal approximation to construct confidence intervals. This requires use of pseudo-values which are the differences between the original discrimination statistic and the jackknife replicate statistics. The standard deviation of the pseudo-values can therefore be used to estimate the confidence interval [15] as follows where * d* is the discrimination statistic, $\:sd\mathrm{=}\sqrt{\frac{\sum\:{\left(d\mathrm{-}{jk}_{i}\right)}^{2}}{n}}$, and $\:d\pm\:\left(1.96\times\:\frac{sd}{\sqrt{n}}\right)$ [19]. 

Other literature [20, 21] encourages reporting the median absolute deviation (MAD) instead of a confidence interval. This is the median of the pseudo-values and describes the typical deviation without assuming the distribution is symmetric – the larger the MAD, the less reliable the discrimination estimate. Alternatively, the interquartile range of the jackknife replicates can be presented akin to the percentile method [22, 23]. The IQR contains the middle 50% of the replicate estimates, with wider ranges indicating greater variability and therefore lower precision/stability of the estimated discrimination measure.

When only a single patient is dropped from the dataset at a time, confidence intervals can be unrealistically narrow [24]. To address this, we additional employed a delete-d (grouped) jackknife, in which larger subsets of subjects were omitted at each iteration. The unit of deletion remains the patient, thereby preserving the within-patient correlation. Two designs are commonly used [24]. In the non-overlapping grouped jackknife, the cohort is partitioned into * G* approximately equal groups of size $\:d\mathrm{=}\frac{n}{G}$. Each replicate omits one group in turn, providing * G* replicate estimates. Alternatively, a random delete-d jackknife draws, at each iteration, a random subset of d subjects to omit. The randomised version allows * G* to be large (e.g. 200–500), which often improves stability of the estimated variance, while the non-overlapping version is computationally simpler and produces exactly G replicates.

Let ${\hat \theta _{\left( {{\rm{ - }}g} \right)}}$ denote the discrimination statistic computed after omitting group * g* where $\:g\mathrm{=}1,\dots\:,G$, and let $\:\overline{\theta\:}\mathrm{=}\frac{1}{G}{\sum\:}_{g\mathrm{=}1}^{G}{\hat \theta _{\left( {{\rm{ - }}g} \right)}}$ represent the mean of the grouped jackknifed replicates. The delete-d jackknife variance is then estimated by:

$$\hat {Var}\left( {\hat \theta } \right){\rm{ = }}{{G{\rm{ - }}1} \over G}\mathop \sum \limits_{g{\rm{ = }}1}^G {\left( {{{\hat \theta }_{\left( {{\rm{ - }}g} \right)}}{\rm{ - }}\bar \theta } \right)^2}$$

and a corresponding 95% confidence interval is given by $\hat \theta \pm \sqrt {\hat {Var}\left( {\hat \theta } \right)}$ where $\hat{\theta\:}$ is the full-sample estimate. This approach is straightforward to implement and has good finite-sample properties when ties reduce the effective sample size [24]. 

### Implementation

Code to evaluate each method of estimating concordance described above can be found at https://github.com/tomjspain/vpmr. This code also enables jackknife resampling allowing for estimation of confidence intervals via all methods described above.

We implemented the core pairwise loop in C++ (Rcpp [25]) which accumulates counts of pair types (e.g. concordant, discordant, tied on predictor, tied on observations) while avoiding pair storage. This keeps runtime 𝑂(𝑛^2^) (jackknife resampling adds an 𝑂(𝑛) pass).

Benchmarks were run in single-threaded mode on an 11th Gen Intel^®^ Core™ i7-11850 H @ 2.50 GHz (8 physical / 16 logical cores) with 63.7 GB RAM, Windows 10 × 64, R 4.4.1 [26], and Rcpp 1.0.14 [25]. The concordance-estimate computation ran in ~ 28.75 s for *n* = 100,000 and ~ 32.72 s for jackknife resampling (~ 10.3% slower), consistent with quadratic scaling.

Additional technical details describing prediction generation for each modelling framework, including mathematical details for estimation of expected recurrent event quantities and cumulative intensity predictions, together with annotated R code illustrating model fitting and estimation procedures, are provided in the Supplementary Material.

### Clinical datasets

Two clinical examples are now presented which show real-life applications of the described methodology. One application is for people with asthma, a chronic lung condition typified by repeated exacerbations, and the other is for people with epilepsy, a neurological condition typified by repeated seizures. People with asthma might have several events per year whilst people with epilepsy might have daily seizures making it a high event rate dataset.

The datasets, eligibility criteria, outcome definitions, and covariates included within the recurrent event prediction models were identical to those used in our previous recurrent event calibration analyses, allowing direct comparison between calibration and discrimination assessment approaches [11]. 

### Asthma – OPCRD

The Optimum Patient Care Research Database (OPCRD) is a large UK-based primary care database comprising anonymised electronic health records from over 600 general practices across England, Scotland, Wales, and Northern Ireland. It has been approved for use in clinical research by the UK Health Research Authority (REC reference: 15/EM/0150) [27]. Full methodology is described elsewhere [11]. 

For this analysis, included patients were those aged 12 to 80 years with a recorded diagnosis of asthma (based on Read codes) prior to study entry and who were registered with a contributing general practice for a minimum of three years. To ensure active asthma, patients were required to have received regular asthma treatment (i.e., more than one prescription excluding short-acting bronchodilators) during the observation period. Individuals with a diagnosis of chronic obstructive pulmonary disease (COPD), a record of resolved asthma, or insufficient follow-up were excluded.

Data were collected between 2005 and 2013, encompassing three consecutive 12-month observation windows per patient. Patients entered the cohort either on January 1st, 2005; upon joining a participating practice; or when their practice commenced data submission to OPCRD.

The outcome of interest was asthma exacerbation, defined in line with European Respiratory Society/American Thoracic Society guidelines [28]. Events were identified as asthma-related hospital admissions, emergency department attendances, or acute consultations resulting in a prescription for oral corticosteroids. Multiple events within a 14-day period were considered part of a single exacerbation episode.

### Epilepsy – SANAD

The Standard and New Antiepileptic Drug (SANAD) studies were two parallel, multicentre, randomised controlled trials evaluating the comparative effectiveness of antiseizure medications. Full study details have been published previously [29, 30]. Arm A focused on individuals with focal epilepsy, while Arm B included those with generalised or unclassifiable epilepsy.

Participants in Arm B were eligible if they had experienced at least two unprovoked epileptic seizures within the previous year and if the treating clinician considered valproate to be the most appropriate standard therapy. Between January 12, 1999, and August 31, 2004, 702 individuals were randomised in a 1:1:1 ratio to valproate, lamotrigine, or topiramate.

The original trial evaluated two primary endpoints—time to treatment failure and time to 12-month remission—using Cox proportional hazards models. For the current analysis, we examined seizure recurrence over a two-year period following randomisation.

### Statistical methods

Two aspects were varied in the analyses. First, we compared several commonly used recurrent event modelling frameworks. Specifically we compared four model types: negative binomial (NB), zero-inflated negative binomial (ZINB), Andersen-Gill (AG), and Prentice, Williams and Peterson – Total Time (PWP-TT). These were chosen in line with a previous publication [11]. The four models were applied to both clinical datasets.

Second, we evaluated four discrimination metrics, Kendall’s τₐ, Goodman-Kruskal’s γ, Somers’ D, and the concordance statistic (c), and estimated uncertainty via jackknifing with normal approximations, with the median absolute deviance and with the interquartile range. We also considered grouped jackknifing in which the patients were partitioned with $\:d\mathrm{=}10$% and $\:G\mathrm{=}200$ [31, 32]. These statistics differ primarily in how they handle tied predictions or outcomes, which occur frequently when event counts are used.

## Results

### Asthma – OPCRD dataset

Results for the asthma dataset are shown in Table 1. The negative binomial (NB) model demonstrated fairly weak discriminatory performance, with a c-statistic of 0.573. Somers’ D and Goodman–Kruskal’s γ were both 0.146, while Kendall’s τₐ was slightly lower at 0.100. The single-case jackknife yielded no variability (MAD = 0; IQR = 0), and the normal-approximation confidence intervals collapsed to the point estimates. In contrast, the grouped jackknife produced non-zero uncertainty (e.g. 95% CI: 0.562–0.584), and the quantile-based intervals were similarly narrow.

**Table 1:** Jack-knifing results for the methods applied to the OPCRD data for people with asthma.$\:{\boldsymbol{\tau\:}}_{\boldsymbol{a}}$,$\:\boldsymbol{\gamma\:}$and D values near 1 indicates strong agreement between predicted risks and observed outcomes, values near − 1 suggest strong disagreement and values close to 0 indicate no association. For the$\:\boldsymbol{c}$ statistic, a value of 0.5 corresponds to flipping a coin to make a prediction for each individual

			Single jackknife				Grouped jackknife	
		Discrim.	95% CI (Normal)	Median	MAD	IQR	95% CI (Wald)	IQR
NB	Kendall	0.100	(0.100–0.100)	0.100	0.000	(0.100–0.100)	(0.085–0.115)	(0.099–0.101)
	Goodman	0.146	(0.146–0.146)	0.146	0.000	(0.146–0.146)	(0.125–0.168)	(0.145–0.148)
	Somers’ D	0.146	(0.146–0.146)	0.146	0.000	(0.146–0.146)	(0.125–0.168)	(0.145–0.148)
	C	0.573	(0.573–0.573)	0.573	0.000	(0.573–0.573)	(0.562–0.584)	(0.572–0.574)
ZINB	Kendall	0.118	(0.118–0.118)	0.118	0.000	(0.118–0.118)	(0.103–0.133)	(0.117–0.119)
	Goodman	0.172	(0.172–0.172)	0.172	0.000	(0.172–0.172)	(0.150–0.193)	(0.170–0.173)
	Somers’ D	0.172	(0.172–0.172)	0.172	0.000	(0.172–0.172)	(0.150–0.193)	(0.170–0.173)
	C	0.586	(0.586–0.586)	0.586	0.000	(0.586–0.586)	(0.575–0.597)	(0.585–0.587)
AG	Kendall	0.442	(0.442–0.442)	0.442	0.000	(0.442–0.442)	(0.431–0.454)	(0.442–0.443)
	Goodman	0.645	(0.645–0.645)	0.645	0.000	(0.645–0.645)	(0.630–0.660)	(0.644–0.646)
	Somers’ D	0.645	(0.645–0.645)	0.645	0.000	(0.645–0.645)	(0.630–0.660)	(0.644–0.646)
	C	0.823	(0.823–0.823)	0.823	0.000	(0.823–0.823)	(0.815–0.830)	(0.822–0.923)
PWP	Kendall	0.603	(0.603–0.603)	0.603	0.000	(0.603–0.603)	(0.594–0.612)	(0.602–0.603)
	Goodman	0.879	(0.879–0.879)	0.879	0.000	(0.879–0.879)	(0.871–0.887)	(0.878–0.879)
	Somers’ D	0.879	(0.879–0.879)	0.879	0.000	(0.879–0.879)	(0.871–0.887)	(0.878–0.879)
	C	0.939	(0.939–0.939)	0.939	0.000	(0.939–0.939)	(0.935–0.944)	(0.939–0.940)

Discrim.=discrimination; MAD = Median absolute deviation; IRQ = Interquartile range

The zero-inflated negative binomial (ZINB) model showed marginally stronger discrimination, with a c-statistic of 0.586. Somers’ D and Goodman–Kruskal’s γ were both 0.172, and Kendall’s τₐ increased slightly to 0.118. As with the NB model, the single jackknife showed no spread, whereas the grouped jackknife produced slightly wider intervals (e.g. 95% CI: 0.575–0.597).

The Andersen–Gill (AG) model yielded considerably higher discrimination, with a c-statistic of 0.823, Somers’ D and γ both at 0.645, and Kendall’s τₐ at 0.442. These values were substantially greater than those for the ZINB model and are indicative of good predictive discrimination. Consistent with the earlier models, the single jackknife showed zero variability, while the grouped jackknife produced a relatively narrow confidence interval (e.g. 95% CI: 0.815–0.830).

Finally, the Prentice–Williams–Peterson Total-Time (PWP-TT) model provided the strongest discrimination overall, with a c-statistic of 0.939. Kendall’s τₐ, Goodman–Kruskal’s γ, and Somers’ D ranged from 0.603 to 0.879. The single jackknife once more showed no variability, whereas the grouped jackknife produced tight confidence intervals (e.g. 95% CI: 0.935–0.944).

### Epilepsy – SANAD dataset

Results for the epilepsy dataset are shown in Table 2. The NB model exhibited moderate discrimination in this dataset, with a c-statistic of 0.654. Confidence intervals obtained using the single-case jackknife collapsed to point estimates for all discrimination measures. In contrast, the grouped jackknife yielded substantially wider and more informative intervals (for example, c-statistic 95% CI: 0.537–0.771; IQR: 0.645–0.663). MAD values for the rank-based measures were small (around 0.001), indicating reasonable stability, although the wider grouped-jackknife intervals suggest greater sampling variability in this dataset than in the previous analysis.

**Table 2:** Jack-knifing results for the methods applied to the SANAD data for people with epilepsy.$\:{\boldsymbol{\tau\:}}_{\boldsymbol{a}}$,$\:\boldsymbol{\gamma\:}$and D values near 1 indicates strong agreement between predicted risks and observed outcomes, values near − 1 suggest strong disagreement and values close to 0 indicate no association. For the$\:\boldsymbol{c}$ statistic, a value of 0.5 corresponds to flipping a coin to make a prediction for each individual

			Single jackknife				Grouped jackknife	
		Discrim.	95% CI (Normal)	Median	MAD	IQR	95% CI (Wald)	IQR
NB	Kendall	0.297	(0.297–0.297)	0.297	0.001	(0.295-0.300)	(0.071–0.524)	(0.282–0.315)
	Goodman	0.307	(0.307–0.307)	0.307	0.001	(0.305–0.310)	(0.074–0.542)	(0.291–0.326)
	Somers’ D	0.307	(0.307–0.307)	0.307	0.001	(0.305–0.310)	(0.074–0.542)	(0.291–0.326)
	C	0.654	(0.654–0.654)	0.654	0.000	(0.653–0.655)	(0.537–0.771)	(0.645–0.663)
ZINB	Kendall	0.298	(0.298–0.298)	0.298	0.001	(0.296-0.300)	(0.073–0.524)	(0.282–0.315)
	Goodman	0.308	(0.308–0.308)	0.308	0.001	(0.306–0.310)	(0.076–0.541)	(0.292–0.326)
	Somers’ D	0.308	(0.308–0.308)	0.308	0.001	(0.306–0.310)	(0.076–0.541)	(0.292–0.326)
	C	0.645	(0.654–0.654)	0.540	0.001	(0.653–0.655)	(0.538–0.771)	(0.646–0.663)
AG	Kendall	0.275	(0.275–0.275)	0.654	0.001	(0.273–0.278)	(0.028–0.524)	(0.258–0.292)
	Goodman	0.285	(0.285–0.285)	0.285	0.001	(0.283–0.287)	(0.029–0.543)	(0.268 = 0.303)
	Somers’ D	0.285	(0.285–0.285)	0.285	0.001	(0.283–0.287)	(0.029–0.543)	(0.268–0.303)
	C	0.642	(0.642–0.642)	0.642	0.001	(0.641–0.644)	(0.514–0.771)	(0.634–0.651)
PWP	Kendall	0.856	(0.856–0.856)	0.856	0.000	(0.855–0.856)	(0.779–0.932)	(0.849 = 0.861)
	Goodman	0.886	(0.886–0.886)	0.886	0.000	(0.885–0.886)	(0.824–0.948)	(0.881–0.890)
	Somers’ D	0.886	(0.886–0.886)	0.886	0.000	(0.885–0.886)	(0.824–0.948)	(0.881–0.890)
	C	0.943	(0.943–0.943)	0.943	0.000	(0.943–0.943)	(0.912–0.974)	(0.940–0.945)

Discrim.=discrimination; MAD = Median absolute deviation; IRQ = Interquartile range

The ZINB model showed discrimination very similar to that of the NB model, with a c-statistic of 0.654 and closely comparable values for Kendall’s τₐ (0.298), Somers’ D (0.308), and Goodman–Kruskal’s γ (0.308). As for the NB model, while the single jackknife showed effectively no variation, the grouped jackknife again produced wide uncertainty ranges (e.g. 95% CI: 0.538–0.771), driven by substantial between-group variability. The MAD values were narrow (approximately 0.001).

The AG model provided slightly weaker discrimination than the NB and ZINB models, with a c-statistic of 0.642, Somers’ D and γ both equal to 0.285, and Kendall’s τₐ equal to 0.275. As before, the single jackknife showed minimal sampling variation, while the grouped jackknife yielded wider intervals (e.g. 95% CI: 0.514–0.771). The spread measures were small (MAD approximately 0.001; IQR values of 0.634–0.651).

In contrast, the Prentice–Williams–Peterson Total-Time (PWP-TT) model again achieved the highest discrimination, with a c-statistic of 0.943. Kendall’s τₐ was 0.856, while both Somers’ D and γ were 0.886. Single-case jackknife intervals were degenerate, but the grouped jackknife produced a 95% CI of 0.912–0.974. The MAD values were minimal (approximately 0) and the quantile intervals were narrow.

**Fig. 1:**
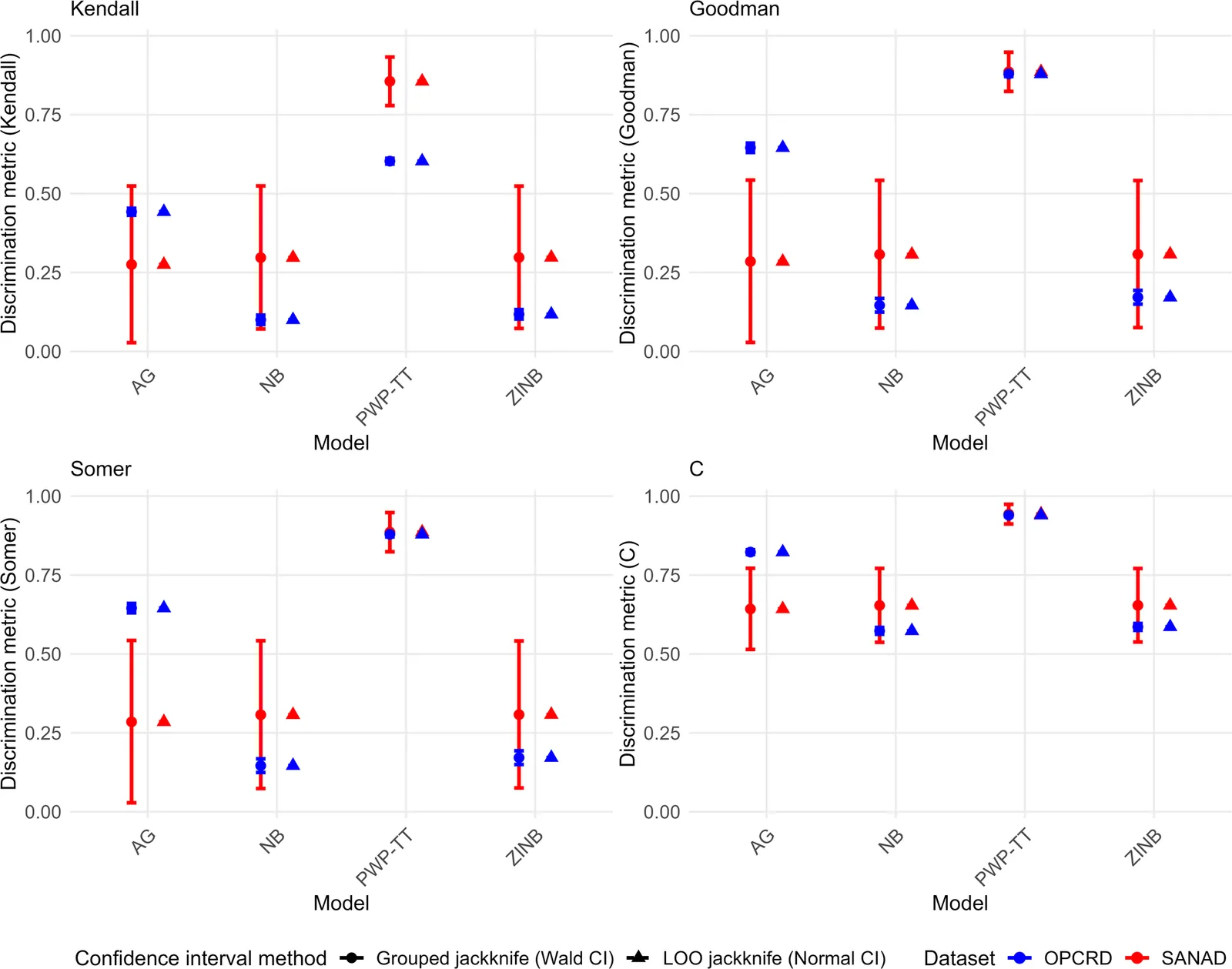
Discrimination estimates with confidence intervals obtained using standard and grouped jackknife methods. Point estimates and 95% confidence intervals for four discrimination measures (Kendall, Goodman, Somers, and C) are shown for each model in the OPCRD (blue) and SANAD (red) datasets. Circles denote estimates with confidence intervals from the grouped jackknife, and triangles denote estimates with confidence intervals from the standard leave-one-out jackknife

Figure 1 discrimination estimates and confidence intervals across models and datasets using standard leave-one-out and grouped jackknife variance estimators. Across all discrimination measures and models, confidence intervals were substantially narrower in OPCRD than in SANAD, including when using the grouped jackknife. Under grouped jackknifing, models with higher discrimination, particularly PWP-TT, tended to have narrower confidence intervals than models with poorer discrimination. In contrast, confidence intervals obtained using the standard leave-one-out jackknife were uniformly small across models and datasets and showed limited sensitivity to differences in discrimination strength or data structure.

## Discussion

This study evaluated the discriminatory performance of several commonly used recurrent event models using a suite of concordance-based statistics adapted for the recurrent outcome setting. In this work, discrimination was evaluated by comparing predicted and observed cumulative event counts within a specified follow-up period. Our findings illustrate clear and substantial differences in discrimination across models. In the two illustrative datasets considered here, the PWP-TT model produced predictions that ranked individuals more accurately than the alternative models examined. However, this should not be interpreted as a general recommendation to prefer PWP-TT models in all settings. Model choice should remain guided by the scientific question, underlying event process, and model fit. The purpose of these comparisons is instead to illustrate how the proposed discrimination measures can reveal differences in predictive ranking performance between models when applied to recurrent event data.

Our proposed discrimination methodology builds on the logic of concordance-based metrics by comparing predicted and observed cumulative event counts within a fixed time horizon for all possible pairs of individuals.

While conceptually similar to the c-index for recurrent events introduced by Kim et al. [33], our approach differs in both scope and implementation. Kim et al. define concordance using subject-specific mean cumulative functions under a proportional rates framework, with inference derived through wild bootstrap techniques [34, 35]. These techniques are useful for heteroskedastic errors and construct bootstrap samples by multiplying the residuals from the original linear model by certain random weights. They have dependent variables that are related to the independent variables by the same linear model [36]. In contrast, we adopt a more flexible framework that does not assume a specific model form and instead focuses directly on the predicted number of recurrent events within a specified time horizon. We include a range of tie-handling strategies (including Kendall’s τ, Goodman-Kruskal’s γ, and Somers’ D), provide openly available R code, and demonstrate real-world application using epilepsy and asthma datasets. This enables our approach to be readily applied across a broad range of recurrent event prediction models and clinical contexts.

In our analyses, the estimates for Goodman–Kruskal’s γ and Somers’ D were identical across all models. This is because both statistics are derived from the same counts of concordant and discordant pairs, differing only in scaling [37]. Kendall’s τₐ, by contrast, is sensitive to the total number of pairs including ties, and therefore produced slightly lower values in all models.

In comparison to the work of Bouaziz [38], our method addresses a fundamentally different performance dimension. Bouaziz focuses on prediction accuracy by introducing a novel Brier-like scoring rule to assess the mean squared error between predicted and actual cumulative event counts. This evaluates calibration or overall predictive accuracy rather than discrimination. Our method complements this by quantifying how well a model separates individuals with higher versus lower risk of recurrent events, irrespective of the absolute prediction error. Together, these approaches provide complementary perspectives on model performance, consistent with recommendations for full validation of prediction models for recurrent outcomes.

This study has several strengths. We provide a comprehensive evaluation of model discrimination for recurrent event data using multiple concordance-based statistics, including our adaptations of Kendall’s τₐ, Somers’ D, Goodman-Kruskal’s γ, and the c-statistic. These metrics offer complementary perspectives on a model’s ability to rank individuals by risk, providing a practical extension of widely used discrimination measures to the recurrent-event setting, moving beyond the limitations of standard measures. Importantly, we evaluated variability using both the standard jackknife and a grouped, delete-d jackknife, allowing us to compare the behaviour of these estimators under different resampling schemes. The standard jackknife intervals were frequently extremely narrow or degenerate, whereas the grouped jackknife produced wider intervals, particularly for the negative binomial and Andersen-Gill models. The choice of deletion size $\:\left(d\right)$ and the number of replicates $\:\left(G\right)$ is ultimately up the researcher; in our study, these were selected based on practical recommendations appropriate for the sample sizes and event distributions in our datasets, balancing computational feasibility with the stability of variance estimates [31, 32]. 

There are also limitations to consider. The analysis was conducted on two datasets, one from asthma and one from epilepsy, to provide examples with different event rates. These illustrative findings may not generalise to other diseases or settings with different characteristics without further investigation. In particular, the relative performance of different modelling approaches may vary depending on event frequency, follow-up duration, and the structure of within-patient dependence. Differences in the underlying modelling target (event burden versus event timing and ordering) may also influence the interpretation of discrimination measures across modelling frameworks.

The applied analyses presented in this work evaluated apparent discrimination performance within the same datasets used for model development and should therefore be interpreted accordingly. Further work assessing internal and external validation performance of recurrent event prediction models remains important, particularly given potential optimism in apparent performance estimates [1]. External validation in recurrent event settings may introduce additional complexities related to differing follow-up durations, censoring mechanisms, evolving treatment pathways, and secular changes in clinical practice. In practice, when validation datasets contain shorter follow-up than development datasets, discrimination assessment may need to be restricted to a common prediction horizon or based on truncated expected recurrent event estimates within the observable follow-up window.

Across all models, the grouped jackknife produced variability that was often substantially larger than that observed with the standard approach, especially for models with weaker discrimination or more heterogeneous predicted risk. This difference may reflect the strong dependence between pairwise comparisons and the nonlinear structure of the metrics. Similar concerns have been raised in broader resampling literature [19, 31]. The difference between the two approaches highlights the importance of the choice of resampling scheme when quantifying uncertainty. However, confidence interval coverage was not evaluated in this study, so it remains unclear which approach provides coverage closer to the nominal level (e.g. 95% for a 95% confidence interval). Simulation studies would be valuable to investigate the coverage and width of confidence intervals obtained using different resampling approach under varying numbers of ties, event frequencies, sample sizes and degrees of heterogeneity in predicted risk [39]. 

Taken together, our results suggest that different modelling approaches can lead to materially different levels of discriminatory performance, and that metrics tailored to the recurrent setting are essential for meaningful comparison. The proposed metrics provide an easily implementable and interpretable framework for assessing model discrimination in this context, and the use of delete-d jackknife resampling provides a flexible approach to quantify uncertainty without relying solely on asymptotic approximations.

We recommend researchers consider their particular research question, data structure, and modelling assumptions alongside the strengths and weaknesses of the various approaches discussed in this research. Importantly, model choice should not be based solely on apparent predictive performance (e.g. discrimination or calibration), but should be driven by the underlying scientific and clinical context in which the model will be applied.

Different recurrent event modelling frameworks address different scientific questions and assumptions regarding the underlying event process, including whether interest lies primarily in cumulative event burden, event timing, event ordering, or dynamically evolving patient risk. More flexible approaches, including multistate and dynamic prediction frameworks such as landmarking, may also be appropriate in some settings and warrant further investigation in relation to discrimination assessment [40]. 

The discrimination measures considered in this work were evaluated within pre-specified prediction horizons, with both predicted and observed recurrent event quantities restricted to the same follow-up window. Consequently, the proposed measures assess a fixed-horizon prediction target analogous to fixed-horizon risk prediction settings in survival analysis, although using cumulative recurrent event burden rather than binary event status [8]. The choice of prediction horizon should therefore be guided by the relevant scientific or clinical context. Future work could further explore discrimination assessment across multiple prediction horizons or within dynamic prediction settings, including landmarking or time-updated recurrent event prediction frameworks.

Additional future work should explore how these discrimination metrics behave under alternative forms of censoring, time-varying covariates, or more complex dependency structures. Nonetheless, our results underscore the importance of evaluating discrimination directly—beyond measures of model fit or calibration—when selecting models for prediction of recurrent clinical events.

## Conclusions

This study illustrates that discrimination performance can vary substantially across commonly used recurrent event models, illustrated in our examples by stronger discrimination from the PWP-TT model. Concordance-based metrics adapted for recurrent events, including Kendall’s τ, Goodman–Kruskal’s γ, Somers’ D, and the c-statistic, offer complementary perspectives on model performance and are straightforward to implement using our publicly available R code.

Our results highlight the value of considering delete-d jackknife resampling when quantifying uncertainty in discrimination measures. Standard jackknife intervals were often very narrow or degenerate, whereas the delete-d jackknife generally produced wider confidence intervals, particularly for models with moderate discrimination or heterogeneous predictions. These findings support considering delete-d jackknife resampling. However, the coverage properties of the jackknife intervals were not evaluated. Future research to determine the coverage of resampling approaches relative to nominal levels under various recurrent event models needs carefully designed simulation studies.

This guide and accompanying code provide practical tools for evaluating discrimination in prediction models for recurrent clinical events. We encourage use of these methods in both model development and external validation studies; however, we recommend that researchers select a small number of appropriate metrics that align with the specific research question, data structure and modelling assumptions, rather than applying all measures simultaneously, to support coherent and interpretable assessment of model performance.

## Supplementary Information

Below is the link to the electronic supplementary material.

Supplementary Material 1

## Data Availability

OPCRD data may be obtained from a third party and are not publicly available.The SANAD datasets analysed during the current study are not publicly available as they contain information that could comprise the privacy of participants but are available from the Professor Marson (mailto: A.G.Marson@liverpool.ac.uk) on reasonable request. R scripts for the analyses described in this manuscript are available from (https://github.com/tomjspain/vpmr).

## Abbreviations

AG: Andersen–Gill
NB: Negative Binomial
OPCRD: Optimum Patient Care Research Database
PWP: Prentice, Williams and Peterson
SANAD: Standard versus New Antiepileptic Drug Study
ZINB: Zero–Inflated Negative Binomial
