# Supplementary material for: Evaluating discrimination of prediction models for recurrent clinical events
Source: Diagn Progn Res. 2026 Sep 7;10:23. doi: 10.1186/s41512-026-00238-7 (PMC13551822; doi:10.1186/s41512-026-00238-7)
Supplement: Supplementary file 1 [file 41512_2026_238_MOESM1_ESM.docx]

# Supplementary Material: Derivation of Predicted Cumulative Event Counts and R Implementation

## S1. Notation and General Framework

Let $i=1,\ldots,n$ index individuals, $t^{*}$ denote the prediction horizon (e.g. 1 year for asthma, 2 years for epilepsy), $\mathbf{x}_{i}$ the covariate vector for individual $i$, and $\hat{\boldsymbol{\beta}}$ the vector of estimated regression coefficients. Where applicable, $t_{ij}$ denotes the time of the $j$th event for individual $i$, and $\delta_{ij}$ the corresponding event indicator.

Across all four models considered in this paper, the key prediction used for discrimination evaluation is the **expected cumulative event count** for individual $i$ up to the prediction horizon $t^{*}$: $\hat{\mu}_{i}\left( t^{*} \right)$. This scalar summary is passed alongside the individual’s observed total event count to the concordance calculations described in the main paper.

## S2. Negative Binomial (NB) Model

### S2.1 Model specification

Let $Y_{i}$ denote the total event count for individual $i$ observed over a follow-up period of length $f_{i}$. The NB model specifies:

$$Y_{i}\mid\mu_{i}\sim\text{NegBin}\left( \mu_{i}, \theta\right)$$

where $\theta$ is the dispersion parameter and the mean is modelled on the log scale with a follow-up offset:

$$\log\left( \mu_{i} \right)=\mathbf{x}_{i}^{\top}\hat{\boldsymbol{\beta}}+log\left( f_{i} \right)$$

### S2.2 Predicted cumulative event count

The predicted cumulative event count for individual $i$ at the prediction horizon is:

$$\hat{\mu}_{i}=f_{i}\cdot exp\left( \mathbf{x}_{i}^{\top}\hat{\boldsymbol{\beta}} \right)$$

This is obtained in R via predict(fit, type = "link") followed by exponentiation, which recovers $\hat{\mu}_{i}$ on the count scale inclusive of the offset. In the OPCRD analysis the outcome was total asthma exacerbations over three years, with $f_{i}$ in days (fupdur). In the SANAD analysis the outcome was total seizure count over two years, with $f_{i}$ in years (totfup).

## S3. Zero-Inflated Negative Binomial (ZINB) Model

### S3.1 Model specification

The ZINB model is a two-component mixture. With probability $\pi_{i}$ (the zero-inflation probability), individual $i$ belongs to a structural-zero class and contributes no events. With probability $1-\pi_{i}$, they are drawn from a NB distribution with mean $\mu_{i}$ as in S2. Both components may depend on covariates; in the analyses reported here the same covariate set was used for both the count and zero-inflation parts.

### S3.2 Predicted cumulative event count

The marginal expected event count, accounting for the zero-inflation mixture, is:

$$\hat{\mu}_{i}^{\text{ZINB}}=\left( 1-\hat{\pi}_{i} \right)\cdot\hat{\mu}_{i}$$

where $\hat{\pi}_{i}$ is the estimated probability of structural zero for individual $i$ (Zeileis, 2008).

In the OPCRD analysis this marginal mean is returned directly by predict(fit, type = "response") from the pscl package, so no further transformation is required. In the SANAD analysis the ZINB was implemented in two stages: a separate NB model was fitted to non-zero counts and a logistic model was fitted for the zero-inflation indicator. The marginal predicted count was then assembled manually as $\left( 1-\hat{\pi}_{i} \right)\cdot\hat{\mu}_{i}$, where $\hat{\pi}_{i}$ was obtained from the logistic part and $\hat{\mu}_{i}$ from exponentiating the count-part linear predictor.

## S4. Andersen–Gill (AG) Model

### S4.1 Model specification

The AG model extends the Cox model to the recurrent event setting by treating each event occurrence as an additional row in a counting-process format (start, stop, status) per patient. A common baseline hazard is assumed across all event occurrences:

$$\lambda_{i}\left( t \right)=\lambda_{0}\left( t \right)\cdot exp\left( \mathbf{x}_{i}^{\top}\hat{\boldsymbol{\beta}} \right)$$

where $\lambda_{0}\left( t \right)$ is the baseline hazard estimated via the Breslow estimator, and cluster-robust standard errors are used to account for within-patient correlation (Andersen & Gill, 1982).

### S4.2 Predicted cumulative event count

Following Therneau & Grambsch (2000, eq. 4.4), the compensator $\hat{E}_{i}(t)$ gives the expected number of events; for fixed covariates over interval $(l,u]$ this reduces to:

$$\hat{m}_{i}(l,u]=exp\left( \mathbf{x}_{i}^{\top}\hat{\boldsymbol{\beta}} \right)\cdot\left[ \hat{\Lambda}_{0}\left( u \right)-\hat{\Lambda}_{0}\left( l \right) \right]$$

where $\hat{\Lambda}_{0}\left( t \right)=\int_{0}^{t} \hat{\lambda}_{0}\left( s \right) ds$ is the Breslow cumulative baseline hazard (Andersen & Gill, 1982). This quantity is returned row-wise via predict(fit, type = "expected").

The total predicted event count up to horizon $t^{*}$ is the sum of these increments over all intervals for individual $i$ with $u\leq t^{*}$:

$$\hat{\mu}_{i}\left( t^{*} \right)=\sum_{j} \hat{m}_{i}(t_{i,j-1}, t_{ij}]$$

In practice this is computed as the cumsum of row-level predictions per patient, taking the value at the last observed interval (which.max(tstop)). Rows with zero-length intervals ($t_{\text{start}}=t_{\text{stop}}$) are excluded before calling predict, as these arise when two events occur on the same day and can produce undefined contributions from the Breslow estimator.

## S5. Prentice–Williams–Peterson Total Time (PWP-TT) Model

### S5.1 Model specification

The PWP-TT model extends the AG framework by allowing the baseline hazard to differ across event strata, where stratum $k$ corresponds to the $k$th event occurrence (Prentice, 1981). Regression coefficients $\hat{\boldsymbol{\beta}}$ are shared across strata:

$$\lambda_{ik}\left( t \right)=\lambda_{0k}\left( t \right)\cdot exp\left( \mathbf{x}_{i}^{\top}\hat{\boldsymbol{\beta}} \right)$$

Separate Breslow cumulative baseline hazards $\hat{\Lambda}_{0k}\left( t \right)$ are estimated for each stratum $k$. Individual $i$ is at risk for stratum $k$ only when exactly $k-1$ prior events have occurred.

### S5.2 Predicted cumulative event count

The expected contribution from stratum $k$ for individual $i$ in interval $(l, u]$ is:

$$\hat{m}_{ik}(l,u]=exp\left( \mathbf{x}_{i}^{\top}\hat{\boldsymbol{\beta}} \right)\cdot\left[ \hat{\Lambda}_{0k}\left( u \right)-\hat{\Lambda}_{0k}\left( l \right) \right]$$

The total predicted event count up to $t^{*}$ sums over both strata and intervals within strata:

$$\hat{\mu}_{i}\left( t^{*} \right)=\sum_{k} \sum_{j} \hat{m}_{ik}(t_{i,j-1}, t_{ij}]$$

As with the AG model, this is computed by accumulating the row-level predict(fit, type = "expected") values via cumsum per patient and taking the value at the last observed interval. The stratum is specified in R via strata(enum) in the coxph formula, where enum indexes the event occurrence number. In the SANAD analysis, predictions were additionally restricted to intervals with stop <= 730 days (the two-year horizon) before accumulation.

## S6. Annotated R Code

The following self-contained code illustrates model fitting and prediction extraction for each of the four model types. Variable names correspond to those used in the analyses described in the main paper (OPCRD dataset shown; the SANAD equivalent is structurally identical with dataset-specific covariate names and a horizon of 730 days).

### S6.1 Data requirements

Two data structures are required: (i) a **person-level** dataset (one row per individual) for the NB and ZINB models, containing the total event count over follow-up (event_count), follow-up duration (follow_up), and covariates; and (ii) a **counting-process** dataset (one row per interval per individual) for the AG and PWP-TT models, with columns Patient_ID, tstart, tstop, status, enum (event occurrence index), and covariates.

### S6.2 Required packages

```
library(MASS)        # glm.nb()
library(pscl)        # zeroinfl()
library(survival)    # coxph()
library(data.table)  # fast per-patient cumsum
```

### S6.3 Negative Binomial

```
# Fit
nb_fit <- glm.nb(
  event_count ~ x1 + x2 + offset(log(follow_up)),
  data = person_dat
)

# Predicted cumulative event count (scaled by individual follow-up via offset)
person_dat$pred_nb <- exp(predict(nb_fit, type = "link"))
```

### S6.4 Zero-Inflated Negative Binomial

When using the pscl package (OPCRD analysis), the marginal expected count $\left( 1-\hat{\pi}_{i} \right)\cdot\hat{\mu}_{i}$ is returned directly:

```
# Fit (count and zero-inflation parts share the same covariate set)
zinb_fit <- zeroinfl(
  event_count ~ x1 + x2 + offset(log(follow_up)),
  data = person_dat, dist = "negbin"
)

# Marginal predicted count: (1 - pi_i) * mu_i, returned directly
person_dat$pred_zinb <- predict(zinb_fit, type = "response")
```

When the two parts are fitted separately (SANAD analysis), the marginal count must be assembled manually:

```
# Step 1: logistic model for P(event > 0)
logist_fit <- glm(I(event_count > 0) ~ x1 + x2 + offset(log(follow_up)),
                  family = binomial, data = person_dat)
prob_nonzero <- predict(logist_fit, type = "response")   # 1 - pi_i

# Step 2: NB model fitted only on non-zero counts
nb_nonzero <- glm.nb(
  event_count ~ x1 + x2 + offset(log(follow_up)),
  data = subset(person_dat, event_count > 0)
)
mu_i <- exp(predict(nb_nonzero, newdata = person_dat, type = "link"))

# Step 3: marginal expected count
person_dat$pred_zinb <- prob_nonzero * mu_i
```

### S6.5 Andersen–Gill

```
# Fit (counting-process format: Surv(tstart, tstop, status))
ag_fit <- coxph(
  Surv(tstart, tstop, status) ~ x1 + x2 + cluster(Patient_ID),
  data = long_dat
)

# Exclude zero-length intervals before predicting
long_dat_nz <- subset(long_dat, tstart != tstop)
long_dat_nz$row_pred <- predict(ag_fit, newdata = long_dat_nz,
                                type = "expected")

# Accumulate per patient; take value at last interval
ag_dt <- data.table(long_dat_nz, key = "Patient_ID")
ag_dt[, pred_ag_cum := cumsum(row_pred), by = Patient_ID]
ag_pred <- ag_dt[, .SD[which.max(tstop), ], by = Patient_ID]
# ag_pred$pred_ag_cum is the predicted total event count per patient
```

### S6.6 Prentice–Williams–Peterson Total Time

```
# Fit (strata() specifies separate baseline hazards per event occurrence)
pwp_fit <- coxph(
  Surv(tstart, tstop, status) ~ x1 + x2 +
    cluster(Patient_ID) + strata(enum),
  data = long_dat
)

# Row-level expected counts (stratum-specific cumulative intensity increments)
long_dat_nz$row_pred_pwp <- predict(pwp_fit, newdata = long_dat_nz,
                                    type = "expected")

# Accumulate per patient; take value at last interval within horizon
pwp_dt <- data.table(long_dat_nz, key = "Patient_ID")
pwp_dt[, pred_pwp_cum := cumsum(row_pred_pwp), by = Patient_ID]
pwp_pred <- pwp_dt[, .SD[which.max(tstop), ], by = Patient_ID]
# pwp_pred$pred_pwp_cum is the predicted total event count per patient
```

### S6.7 Passing predictions to vpmr

Once person-level predicted counts have been obtained from any of the four models above, they are passed alongside the observed total counts to the vpmr package for discrimination evaluation:

```
# Install from GitHub if required
# remotes::install_github("tomjspain/vpmr")
library(vpmr)

# Example using NB predictions
result <- vpmr(
  predicted = person_dat$pred_nb,
  observed  = person_dat$event_count
)
print(result)   # returns c, tau_a, Goodman-Kruskal gamma, Somers' D
                # with jackknife confidence intervals
```

Full documentation, including grouped (delete-$d$) jackknife options, is available at <https://github.com/tomjspain/vpmr> and the accompanying GitHub Pages site at <https://tomjspain.github.io/vpmr>.

## References

- Andersen PK, Gill RD (1982). Cox's regression model for counting processes: a large sample study. *Annals of Statistics*, 10(4): 1100–1120.
- Prentice RL, Williams BJ, Peterson AV (1981). On the regression analysis of multivariate failure time data. *Biometrika*, 68(2): 373–379.
- Therneau TM, Grambsch PM (2000). *Modeling Survival Data: Extending the Cox Model*. Springer, New York.
- Zeileis A, Kleiber C, Jackman S (2008). Regression models for count data in R. *Journal of Statistical Software*, 27(8): 1–25.
